# Adsorption of 4-(*N*,*N*-Dimethylamino)-4′-nitrostilbene on an Amorphous Silica Glass Surface

**DOI:** 10.1021/acs.jpcc.3c05552

**Published:** 2023-11-17

**Authors:** Dóra Vörös, Andrea Angeletti, Cesare Franchini, Sebastian Mai, Leticia González

**Affiliations:** † Institute of Theoretical Chemistry, Faculty of Chemistry, University of Vienna, Währinger Straße 17, 1090 Vienna, Austria; ‡ Computational Materials Physics, Faculty of Physics, University of Vienna, Kolingasse 14-16, 1090 Vienna, Austria; § Vienna Doctoral School in Physics, University of Vienna, Boltzmanngasse 5, 1090 Vienna, Austria; ∥ Department of Physics and Astronomy ’Augusto Righi’, Alma Mater Studiorum—Università di Bologna, Bologna 40127, Italy

## Abstract

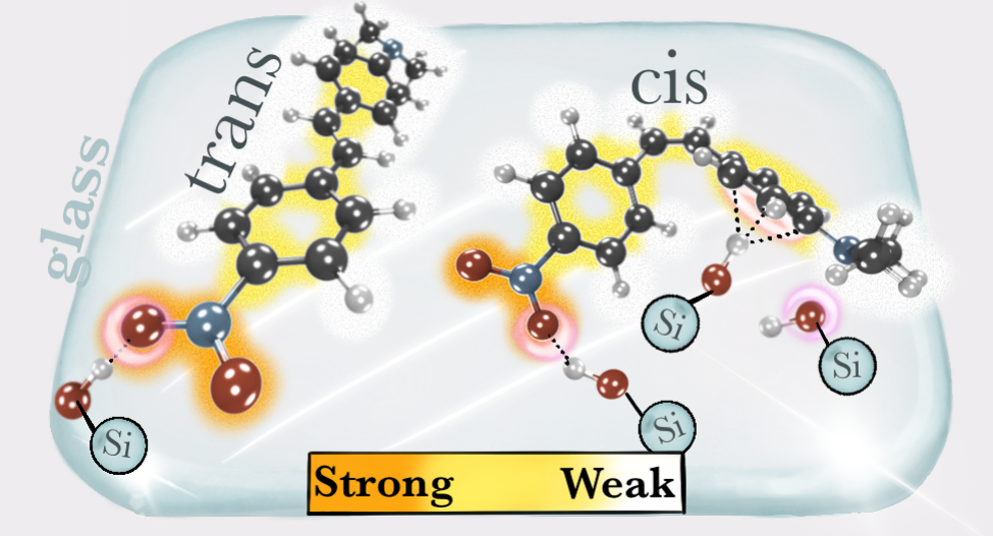

Stilbenes are a compelling class of organic photoswitches with a high degree of tunability that sensitively depend on their environment. In this study, we investigate the adsorption properties of 4-(*N*,*N*-dimethylamino)-4′-nitrostilbene (DANS), a push–pull stilbene, on amorphous silica glass. Plane-wave density functional theory (DFT) calculations are used to understand how the *trans* and *cis* isomers of DANS interact with the amorphous surface and which are the most preferred modes of adsorption. Our calculations revealed that the O–H···O hydrogen bonds between the nitro group and hydroxyl groups of the silica surface dominate the intramolecular interaction. In addition to hydrogen bonding, O–H···π interactions with the aromatic ring and double bond play a critical role in adsorption, whereas C–H···O interactions are present, but contribute little. Therefore, both isomers of DANS favor parallel orientations such that not only the functional groups but also the aromatic parts can strongly interact with the glass surface.

## 1 Introduction

Stilbene-based photoswitches have been subject to extensive research in the past^1−6^ as well as are nowadays^7−9^ due to their wide applicability.^10−16^ They constitute a versatile scaffold where both fluorescence and nonradiative isomerization can be modulated by the incorporation of functional groups.^17^ Push–pull stilbenes, featuring both an electron donor and an electron acceptor moiety, are particularly garnering attention due to their electron coupling properties between the donor and acceptor groups.^9^ Upon photoexcitation, the donor–acceptor coupling can lead to significant charge transfer (CT) character in the electronic excited states. Increasing the push–pull effect leads to greater charge separation and twisted intramolecular CT, as demonstrated in previous investigations.^18,19^

**Figure 1:**
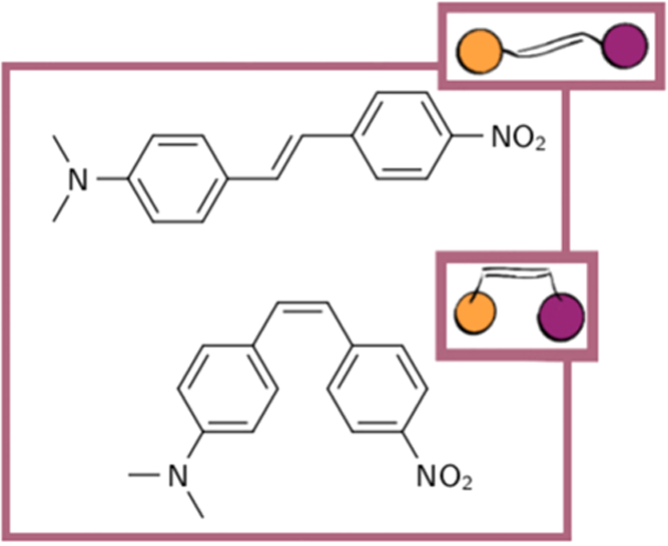
*Trans*- and *cis*-4-(*N*,*N*-dimethylamino)-4′-nitrostilbene (DANS), together with simplified representations as used below. The purple circle symbolizes the aromatic ring with the nitro group, while the orange circle stands for the aromatic ring with the dimethylamino functional group.

One interesting example of a strong push–pull stilbene derivative is 4-(*N*,*N*-dimethylamino)-4′-nitrostilbene (DANS, see Figure 1).^17,20^ The dipole moment of DANS changes significantly from the electronic ground to the excited states, making its emissive and nonradiative properties rather sensitive to its surrounding.^17,19^ For example, DANS exhibits significant fluorescence, with a yield of about 50%^21^ in nonpolar solvents such as benzene and toluene, but its emission decreases significantly in solvents with strong polarity due to the interactions with the polar CT state.^21^ Furthermore, recent studies^11^ found alterations in the fluorescence emission when DANS interacts with various microplastics composed of polymers with different polarities, making DANS a potential tool to detect microplastics in water.

Another molecular environment that exhibits noteworthy interactions with photoswitches like DANS (and also with nitro compounds in general) is amorphous silica (SiO_2_).^22−25^ This material has interesting surface properties and is therefore also frequently used, e.g., in chromatography,^26^ catalysis,^27,28^ medicine,^28−30^ or chemical sensing.^31−33^ These diverse applications are enabled by the presence of a large number of hydroxyl groups on the surface, which can interact with each other as well as with adsorbates, in particular through hydrogen bonds (HB).^34,35^ Due to the nature of amorphous silica, the density of the hydroxyl groups varies significantly, leading to denser patches where surface–surface HB networks are formed and more sparse patches where these networks are disrupted.^35^ Consequently, the adsorption of molecules on amorphous silica surfaces depends critically on the local morphology, as surface–surface HBs (but also other interactions) can compete with surface–adsorbate HBs.^36^

The aim of this paper is to examine the adsorption behavior of DANS on the surface of amorphous silica, given its remarkable sensitivity to the environment^11^ and the broad usage of silica as a carrier material for photoswitches.^22^ We investigate the most stable adsorption geometries of DANS in both conformations (*cis* and *trans*) and unravel the specific interactions that govern the adsorption modes. For this purpose, we optimized several initial orientations of both isomers on four independently sampled configurations of amorphous silica surfaces. We calculated the interaction energies of the optimized structures and analyzed the different types of interactions of DANS with the surface by calculating charge density differences and performing regression analysis. Disentangling the interactions between DANS and the amorphous silica surface holds potential to understand the photoswitching and emissive properties of DANS and related compounds on polar surfaces carrying hydroxyl groups.

## 2 Methods

Our general computational approach consists of generating different orientations of the *cis* and *trans* isomers of DANS, combining them with different glass surface configurations to form initial guesses for the adsorption geometries, and optimizing all of these geometries. The following section provides computational details for these steps, as well as for the subsequent data analysis.

### 2.1 Glass Surface Model and Adsorption Geometries

Studying amorphous surfaces at atomistic scale is computationally challenging, due to the disorder of the system.^37^ Here, we employ a surface model taken from the work of Johnson and co-workers.^38^ In order to describe the randomness of the surface and the surface hydroxyl groups, we consider multiple surface structures. In particular, we chose two glass slabs from previous work^38^—which were obtained at 9 and 56 °C, respectively—and employ the top and bottom sides of each slab, making a total of four surface models (labeled A/B and C/D for the top/bottom sides at each temperature, respectively). Note that the two slabs have different numbers of atoms. Both slabs were relaxed with plane-wave density functional theory (DFT), as described below, before the initial glass–DANS geometries were prepared.

**Figure 2:**
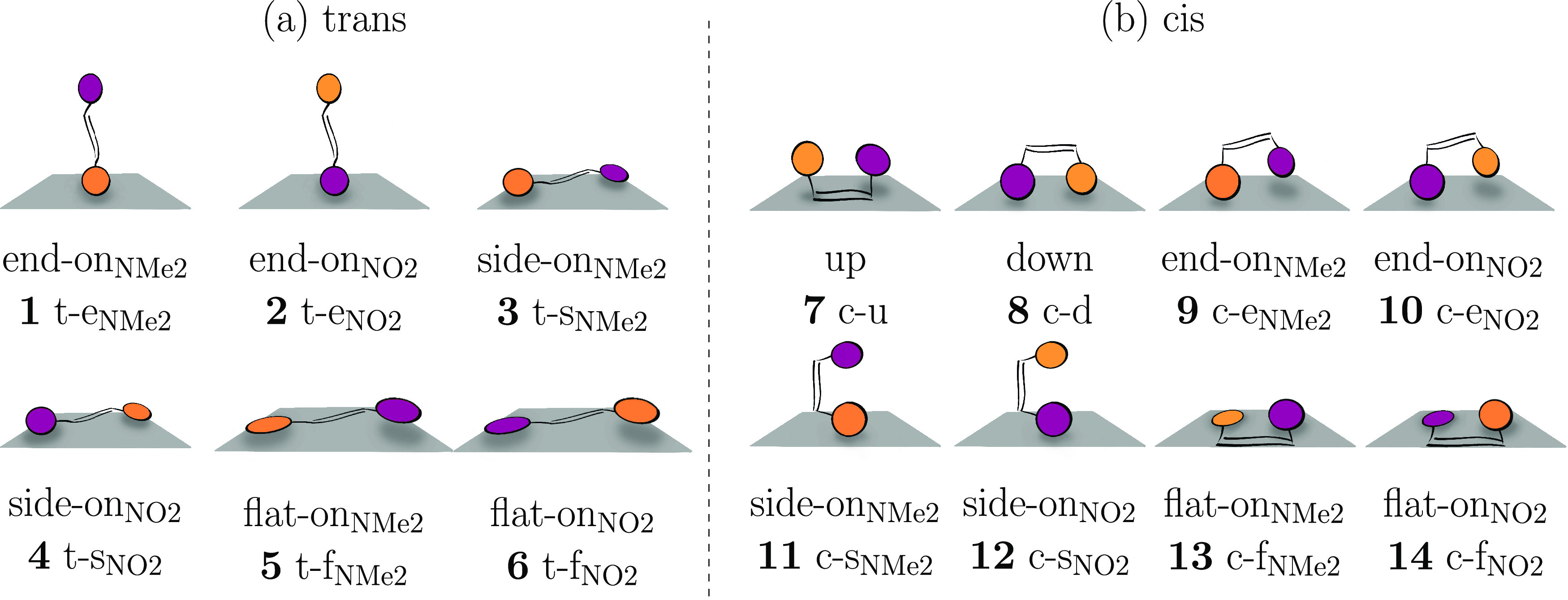
Starting orientations of *trans*-DANS (a) and *cis*-DANS (b) adsorbed on the glass surface. Purple and orange moieties in DANS represent aromatic rings bearing the NO_2_ and NMe_2_ functional groups, respectively.

In order to find realistic adsorption geometries of DANS on the amorphous glass surfaces, we carried out a large set of optimizations, starting from various initial geometries that consider different orientations of DANS. The *cis* and *trans* isomers of DANS were preoptimized at second order Møller–Plesset perturbation theory (MP2)/cc-pVDZ^39^ level of theory with Gaussian 16.^40^ Further details about the choice of the level of theory for the preoptimization can be found in the Supporting Information in Section S1 and Table S1. Subsequently, the two isomers were placed in the center of the unit cell, a few Angstrom above (2.5–4.0 Å) the glass surface, in different orientations to form the various initial geometries depicted in Figure 2. For *trans*-DANS (Figure 2a), we consider six orientations, which correspond to the six faces of a cuboid encasing the oblong molecule—such that every side of the molecule can make contact to the surface. These orientations (**1**–**6**) are labeled as follows: *trans*-end_NMe2_ and *trans*-end_NO2_, where only the indicated functional group is near the surface; *trans*-side_NMe2_ and *trans*-side_NO2_, where some of the hydrogen atoms of the aromatic rings are near the surface; and *trans*-flat_NMe2_ and *trans*-flat_NO2_, where the named aromatic ring is parallel to the surface. We note that the preoptimized *trans*-DANS is not completely planar, but slightly twisted, so that only one of the two rings can be oriented parallel to the glass surface in the flat-on orientations. For the *cis* isomer (Figure 2b), a total of eight orientations were considered (numbered **7**–**14**). Compared to the two end-on structures of *trans*-DANS, in *cis*-DANS, we consider four upright orientations: up (no functional group near the surface), down (both groups near the surface), and end-on (one group near the surface, analogous to the *trans* case). Additionally, we consider two side-on and two flat-on cases for *cis*-DANS, analogous to the case of *trans*-DANS.

The six orientations of *trans*-DANS and the eight orientations of *cis*-DANS give a total of 14 different orientations. As we have four surfaces (A–D) for each orientation, a total of 56 optimizations were performed, labeled from A1 to D14. The orientation labels in Figure 2, e.g., t-e_NMe2_ or c-f_NO2_ are used both to indicate the initial geometries as well as to discuss the obtained optimized structures.

### 2.2 Density Functional Theory Calculations

The 56 prepared initial structures (DANS plus glass) were subsequently optimized using plane-wave DFT with periodic boundary conditions, as implemented in VASP 6.2.1^41−43^ with the projector-augmented wave method.^44^ We employed the Perdew–Burke–Ernzerhof (PBE) exchange-correlation functional and the D3 dispersion scheme with Becke–Johnson damping function.^45,46^ The performance of D3-PBE in describing the adsorption of small molecules on silica has been benchmarked against (experimental) reference values by different authors^47−49^ and this approach was also applied in several further computational studies.^23,50−53^ To obtain an estimate for the possible energy uncertainty due to the functional choice, we applied the optimization process to four structures using the revised Perdew–Burke–Ernzerhof (RPBE)^54^ functional. The results of the comparison of PBE and RPBE are shown in Table S2, showing that the interaction energies agree to within 0.1 eV.

The unit cells of the glass slabs^38^ had dimensions of 23.74 Å × 18.28 Å. The thickness of the glass slabs was about 7–10 Å, and *trans*-DANS is about 15 Å in length. Thus, in the direction perpendicular to the surface, we chose a box length of 40.45 Å, giving a minimum of 15 Å of separation between the molecule and the glass slab in the neighbor cell. The energy cutoff for the plane-wave basis was set to 400 eV. We used Gaussian smearing with a smearing width of 0.05 eV to improve the electronic (self-consistent field) convergence and avoid numerical problems. The electronic convergence threshold was 10^–6^ eV (3.7 × 10^–8^ Hartree).

During the optimizations, all atoms (DANS and glass) were allowed to move, except five Si atoms in the middle of the slab, which were held stationary to maintain the integrity of the glass slab. The ionic (geometry) optimization convergence threshold was set to 0.01 eV/Å (1.9 × 10^–4^ Hartree/Bohr). The structures were fully relaxed by using the conjugate gradient algorithm. For the optimization, the *k*-space was sampled by a Γ-centered 1 × 1 × 1 *k*-mesh (i.e., only the Γ point). This *k*-mesh in combination with Gaussian smearing was found to lead to converged energies (with an uncertainty of no more than 20 meV) for the amorphous insulator system^55^ we are dealing with, as shown in Table S3. Optimized geometries were visualized with Visual Molecular Dynamics (VMD)^56^ and are available online.^57^

For the analysis of the energies and electron densities, we performed additional single-point calculations at the converged geometries. These were carried out with a (3 × 3 × 1) *k*-mesh.

### 2.3 Data Analysis

#### 2.3.1 Geometric Clustering

In order to categorize the 56 structures (see Figures 1 and S2) obtained from the optimizations, we performed K-Means clustering^58^ using scikit-learn in Python.^59^ We employed a set of four geometry parameters: the mean of the two angles between the aromatic rings and the mean plane of the glass surface, the two distances of the two functional groups from the surface, and the mean distance of the two bridge carbon atoms from the surface. The mean distance of the bridge carbon atoms from the surface was only used for the *cis* isomer. These geometry parameters are defined in Section S2.1. The values of these parameters for all 56 structures are listed in Tables S4 and S6 in the SI. The SI also includes a schematic (Figure S3) that illustrates the data we gathered for the clustering.

These data were standardized by centering them around their mean and scaling them to have unit variance. We ran the K-Means algorithm separately for the *trans* and *cis* structures, with *k* clusters chosen via the Elbow method.^60,61^ For each isomer, K-Means was run multiple times with different initializations (randomly spreading the centroids across the data points), selecting the final solution with the lowest variance.

We also performed a principal component analysis (PCA) with the standardized data, selecting the two principal components with the largest variance for visualization of the data. Further details regarding the PCA are shown in Tables S5 and S7.

#### 2.3.2 Energetics

The stability of the different obtained structures is assessed with the help of relative energies (Δ*E*_rel_), evaluated separately for each combination of a glass slab (either A/B or C/D) and an isomer (either *cis* or *trans*). The lowest energy for each slab-isomer combination (*cis*-A/B, *cis*-D/C, *trans*-A/B, and *trans*-C/D) was taken as the respective reference energies. Energies are not compared between different slab-isomer combinations.

Interaction energies (Δ*E*_int_) are calculated as the difference between the total single-point energy of the full system (*E*^system^) and single-point energies of only the glass (*E*_SP_^glass^) and only the DANS (*E*_SP_^DANS^)
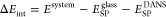
1The single-point calculations are performed on glass and DANS geometries taken from the full-system optimization; i.e., their geometries are not individually relaxed. Note that for plane-wave basis sets, it is not necessary to consider a basis set superposition error.

In contrast, adsorption energies (Δ*E*_ads_) are computed from individually relaxed geometries of the full system, the glass, and DANS
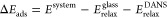
2We note that *E*_relax_^DANS^ was not computed with the preoptimized MP2/cc-pVDZ geometry, but with a geometry optimized at the DFT level of theory, as described in Section 2.2. Compared to the interaction energies, the adsorption energies include the effect of geometric deformation. This effect is directly quantified by computing the deformation energies as Δ*E*_def_^glass^ = *E*_relax_^glass^ – E_SP_^glass^ and Δ*E*_def_^DANS^ = *E*_relax_^DANS^ – *E*_SP_^DANS^. All of the obtained energies of the orientations are listed in Tables S8 and S9. We plotted the interaction energies against the relative energies and the adsorption energies to assess the correlation between them (Figure S4).

#### 2.3.3 Interaction Analysis

In order to identify the effect of adsorption on the electronic structure, we computed charge density differences as
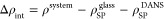
3using VASPKIT 1.3.3.^62^ The charge density differences allow visualizing (using VMD^56^) the electron flow that arises from the interaction between the two fragments and, consequently, to determine the nature of the adsorbant–adsorbate bonding (e.g., HB or dispersion). Additionally, we quantified the electron flow by determining Bader charges^63^ using the Bader charge analysis code.^64^

#### 2.3.4 Multiple Linear Regression Analysis of Interaction Energies

Finally, we sought to quantify the interaction energy contributions arising from contacts of the various parts of the molecule with the glass surface. To this end, we perform a multiple linear regression analysis (with zero intercept) with the R software to model the relationship between the interaction energy (the dependent variable) and the number of molecule–surface contacts (the independent variables). We divided the molecule into six fragments: NO_2_, C_6_ (ring near NO_2_), C_2_ (bridge), C_6_ (ring near NMe_2_), NMe_2_, and all nonmethyl H atoms (see Figure S5). For each fragment, we define suitable distance and angle criteria to quantify the number of contacts to the glass surface (Tables S10 and S11). For HB (of the NO_2_ and NMe_2_ fragments), we used criteria from elsewhere.^65,66^ For the other contact types, we validated the employed distance-angle criteria using the charge density difference plots.

## 3 Results and Discussion

### 3.1 Geometric Clustering

**Figure 3:**
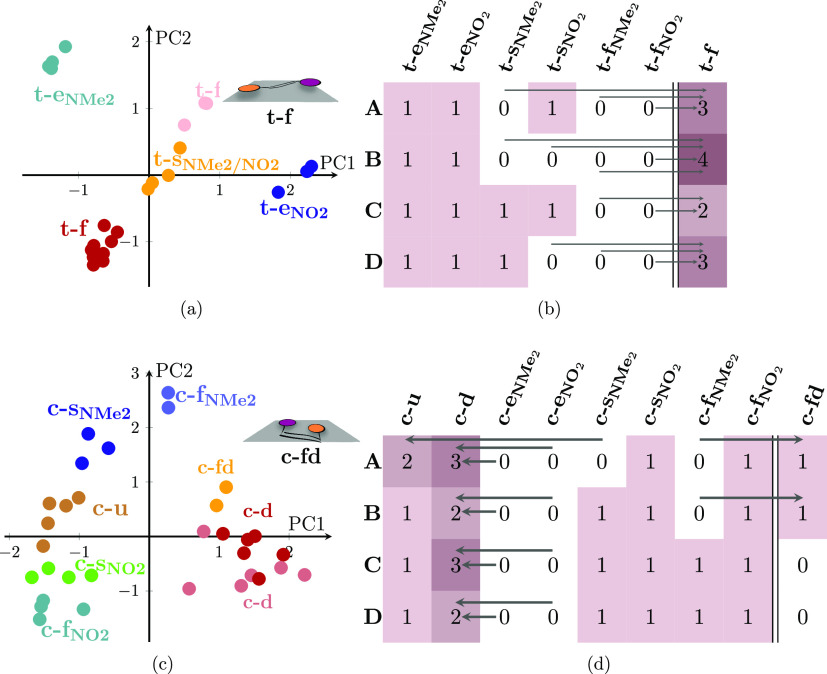
Clustering of the optimized structures of DANS on a glass surface. (a, c) Results of a K-Means clustering and a PCA. See Tables S4 and S6 for the geometric parameters used and Tables S5 and S7 for further information on the principal components (PC1 and PC2). (b, d) Relationship between initial geometries (Figure 2) and optimized geometries. The numbers in the cells indicate how many optimized structures of each orientation were obtained, for each of the four surfaces A–D. Arrows indicate which initial orientations evolved into other optimized orientations. Columns after the double line refer to orientations that have newly emerged after optimization. Schematic representations of the new orientations (t-f and c-fd) are depicted together with the clustering results in (a, c), respectively.

The results of the K-Means clustering analysis of the optimized structures of *trans*-DANS on the four different surfaces (6 orientations × 4 surfaces, i.e., 24 structures) are summarized in Figure 3a. All of the optimized structures for the *trans* isomer are shown in Figure S4 in the SI. We used three out of the four geometric parameters mentioned above for clustering (see Table S4). The results are represented in the space of the two most important principal components, PC1 and PC2. As it can be seen, the clustering gives five well-separated clusters that are assigned to the indicated orientations. This is in contrast to the six initial orientations that we started the optimizations with—not all initial orientations survived and a new one arose, as discussed below.

The most important geometry parameters that distinguish the clusters can be obtained from the loadings of the principal components (Table S5). PC1 correlates mostly with the difference between the distance of the nitro group from the surface and the distance of the dimethylamino group from the surface, as this difference allows the two end-on orientations to be distinguished from each other and from the side-on and flat-on orientations. PC2 then correlates mostly with the average ring–surface angle, which differentiates the flat-on and side-on structures. Note that due to the convention used for the ring–surface angles, there are two flat-on clusters that correspond formally to a 0° ring–surface angle and a 180° angle, respectively (with the side-on geometries in between around 90°). Considering that *trans*-DANS flattens on the glass, these two flat-on clusters are actually indistinguishable. Hence, we use the same label “t-f” for both, which describes the orientation where DANS is lying flat on the surface (schematic representation in Figure 3a), irrespective of which of the two rings is more parallel to the surface (see average angles in Table S1). Due to the ruggedness of the glass surfaces, t-f also includes some geometries where DANS is not perfectly parallel to the mean plane of the surface.

Figure 3b summarizes which of the initial orientations of DANS on the different surfaces survived through the optimizations. The arrows indicate those initial orientations that converged to different final orientations. In the initial geometries, DANS had a twisted structure; thus, we could differentiate which ring is parallel to the surface. However, after the optimization flattens DANS on the surface, the two flat-on orientations (t-f_NO2_ and t-f_NMe2_) become indistinguishable, and hence we apply a new label “t-f”, as mentioned above. Therefore, in Figure 3b, an extra column (after the double line) is used for this orientation.

Starting from the left in Figure 3b, all end-on orientations were preserved by the optimization. We assume that this is due to the presence of very small gradients because all parts of the molecule, except one of the two functional groups (either NO_2_ or NMe_2_), are very far away from the surface (see Table S4 for the end-on orientations). The optimizations of all other orientations (side-on and flat-on) tended to bring the molecule into the t-f (general flat-on) orientation. For the side-on initial orientations, this means that the molecule tends to topple over to increase the contact area and thus the interactions with the glass. In summary, four side-on orientations were preserved, and a total of 12 flat-on orientations were obtained, meaning that these seem to be preferred over side-on orientations. The optimizations also indicate that end-on and flat-on orientations should be (meta-) stable. Yet, since our set of initial orientations might be limited, we do not want to draw general conclusions about the most stable orientations. This would require proper sampling obtained through adsorption dynamics of DANS on the various glass surfaces, which is computationally unfeasible with our chosen electronic structure method.

In Figure 3c, we show the clustering results for the *cis* isomer using four geometric parameters (Table S6). All of the optimized structures for the *cis* isomer are shown in Figure S2 in the SI. A total of eight clusters were identified by the Elbow method. These clusters are arranged in two superclusters. One is located on the left side of the plot, consisting of a continuum that stretches from c-f_NO2_ to c-s_NO2_, c-u, c-s_NMe2_, to c-f_NMe2_. The second supercluster is on the right of the plot, consisting of the flat-on (“c-fd”, new orientation, see schematic in Figure 3c) and two down (c-d) clusters. The two superclusters can be better understood by inspection of the first principal component PC1 (Table S7). Essentially, the left supercluster includes all orientations where at most one functional group is in contact with the glass. Conversely, the right supercluster contains all orientations where both functional groups interact with the surface. PC2 then describes which of the two functional groups is closer to the surface.

While the clustering of the *trans* isomer was relatively straightforward to interpret, the clustering for the *cis* isomer shows three pecularities. First, it shows two separate clusters that we both labeled as “down” because they show the two functional groups pointing toward the surface, although in one cluster DANS is oriented perpendicular to the surface, whereas in the other it is slightly tilted (see mean angles in Table S6). The clustering model we used is able to differentiate between these perpendicular and tilted-down structures, yet we still regard them as c-d orientations (they are not tilted enough to be classified as flat-on). Second, within these two “down” clusters, we found two structures where one of the functional groups is slightly farther away from the surface than the other functional group, making them mixtures between down (both functional groups close to glass) and end-on orientations (one functional group very far from glass). The distances of the functional groups from the surface can be seen in Table S6. Third, besides the two idealized, initially prepared flat-on orientations (c-f_NO2_, c-f_NMe2_), we also obtained a third flat-on orientation that is distinguished by having both functional groups near the surface. This third flat-on orientation is rather separated from c-f_NO2_ and c-f_NMe2_ in the clustering, so we give it a separate label, “c-fd” (see schematic representation in Figure 3c).

Analogous as in *trans*, Figure 3d summarizes the result of the optimizations for the *cis* isomer and which orientations were preserved or converged to another one. For the *cis* isomer, we introduced one extra label, “c-fd”, which is shown in the rightmost column. As indicated by the arrows, all end-on orientations converged toward down orientations. Otherwise, most initial orientations were preserved, with three exceptions (which changed to c-u or c-fd) where the molecule moved slightly to increase the contact area with the surface. In summary, we find that end-on orientations of the *cis* isomer are disfavored due to the small number of contacts with the surface. However, we found realizations of all other orientations, indicating that there are many possible ways to adsorb the irregularly shaped *cis*-DANS on the rugged glass surfaces. The only clear preference seems to be to have a large number of contacts.

In conclusion, we have found that both the *trans*- and *cis*-DANS disfavor orientations with few contacts. As we see in the *cis* case, end-on orientations are not stable, which we assume should also be true for the *trans* isomer, even though our optimizations lead to (meta-) stable end-on structures. Conversely, all other orientations seem to be principally feasible. Since the geometrical clustering analysis does not provide information about the relative stability of the orientations, we will discuss energetics in the next section.

### 3.2 Energetics

**Figure 4:**
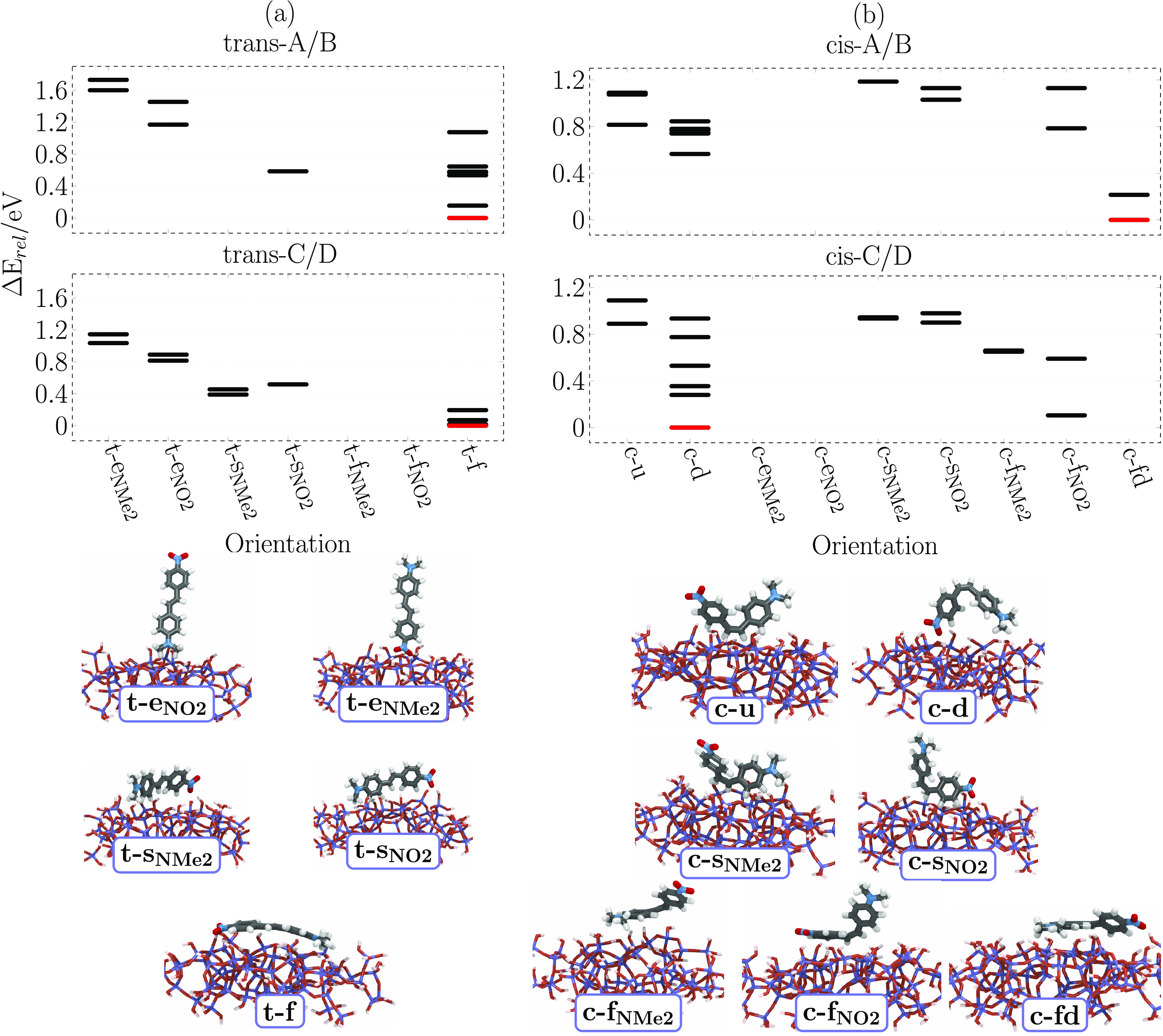
Relative energies of the optimized *trans* (a) and *cis* (b) isomers. The relative energies are shown separately for the different slab-isomer combinations and are grouped according to the clustering analysis in Figure 3. The reference energies are represented in red. Below the energy plots, representative structures of each obtained orientation are depicted.

Next, we investigated the energetic differences between the obtained structures. Figure 4 depicts the relative energies of the *trans* (panel a) and *cis* (panel b) isomers on the different glass slabs. Each slab-isomer combination is shown in a separate plot, as their energies are not comparable. All relative energies are collected in Tables S8 and S9.

For the *trans* isomer (Figure 4a), the following energetic order is found: t-e_NMe2_ > t-e_NO2_ > t-s_NMe2/NO2_ ∼ t-f. Thus, the end-on orientations are the least favored ones, with energies that are >1 eV higher than those of the most stable side-on and flat-on structures. For both glass slabs, the lowest energy was found for the t-f orientation. However, this orientation presents a significant energy spread, so although it shows one system with the lowest energy, other t-f structures also show very high energies. Upon closer inspection, we find that this energy spread is due to a varying number of contacts or strength of bonding of the molecule with the glass due to the ruggedness of the surfaces and the random distribution of the hydroxyl groups. The side-on and flat-on orientations seem favorable because in general they permit a larger number of contacts to be formed compared to the end-on orientations. This can be appreciated in the molecular structures depicted at the bottom of Figure 4a.

For *cis*, the energetic ordering (Figure 4b) is c-u ∼ c-s_NMe2/NO2_ > c-f_NMe2/NO2_ ∼ c-d > c-fd. Recall that the relative energies between the two plots should not be compared; e.g., c-fd in the upper plot and c-d in the lower one both have relative energies of 0 eV, but c-fd is always below c-d wherever it appears. Generally, orientations with both functional groups in contact with the surface tend to have a low energy. Besides c-d, also c-fd exhibits these two contacts, which is precisely what differentiates c-fd from the other flat-on orientations (c-f_NO2_, c-f_NMe2_), see bottom structures in Figure 4b. For c-d, we observe a wide range of relative energies, again attributed to the ruggedness of the surfaces and the consequent randomness in the available hydroxyl groups. Even though c-fd was only obtained two times, it appears to be a very stable adsorption configuration due to the large contact area that it affords. Perhaps surprisingly, the c-u orientation has comparable energy to that of the side-on orientations, even though c-u does not exhibit any interactions of the functional groups with the surface.

We note that the conclusions drawn from these results should be regarded as qualitative trends. A rigorous quantitative investigation of the relative adsorption energies of the different orientations would require extensive sampling of both the surface and the orientation of DANS with expensive ab initio molecular dynamics simulations, beyond the scope of this work. However, the present results—obtained by sampling four different glass surfaces—do indicate that low relative energies are correlated with structures where (optimally) both functional groups and the conjugated system are in contact with the surface.

### 3.3 Analysis of Interactions

Previous research^34,35^ on molecules adsorbed on silica surfaces pointed out the critical role of the formation of HBs between the hydroxyl groups of the glass and the adsorbate. Polar functional groups like nitro groups^23−25^ form particularly strong interactions, resulting in tightly adsorbed molecules. However, the results of the previous sections indicate that the HBs of the functional groups with the surface are not the only interactions that govern the adsorption configuration of DANS. Thus, we next discuss the individual interactions and contacts between the adsorbate and the glass substrate.

**Figure 5:**
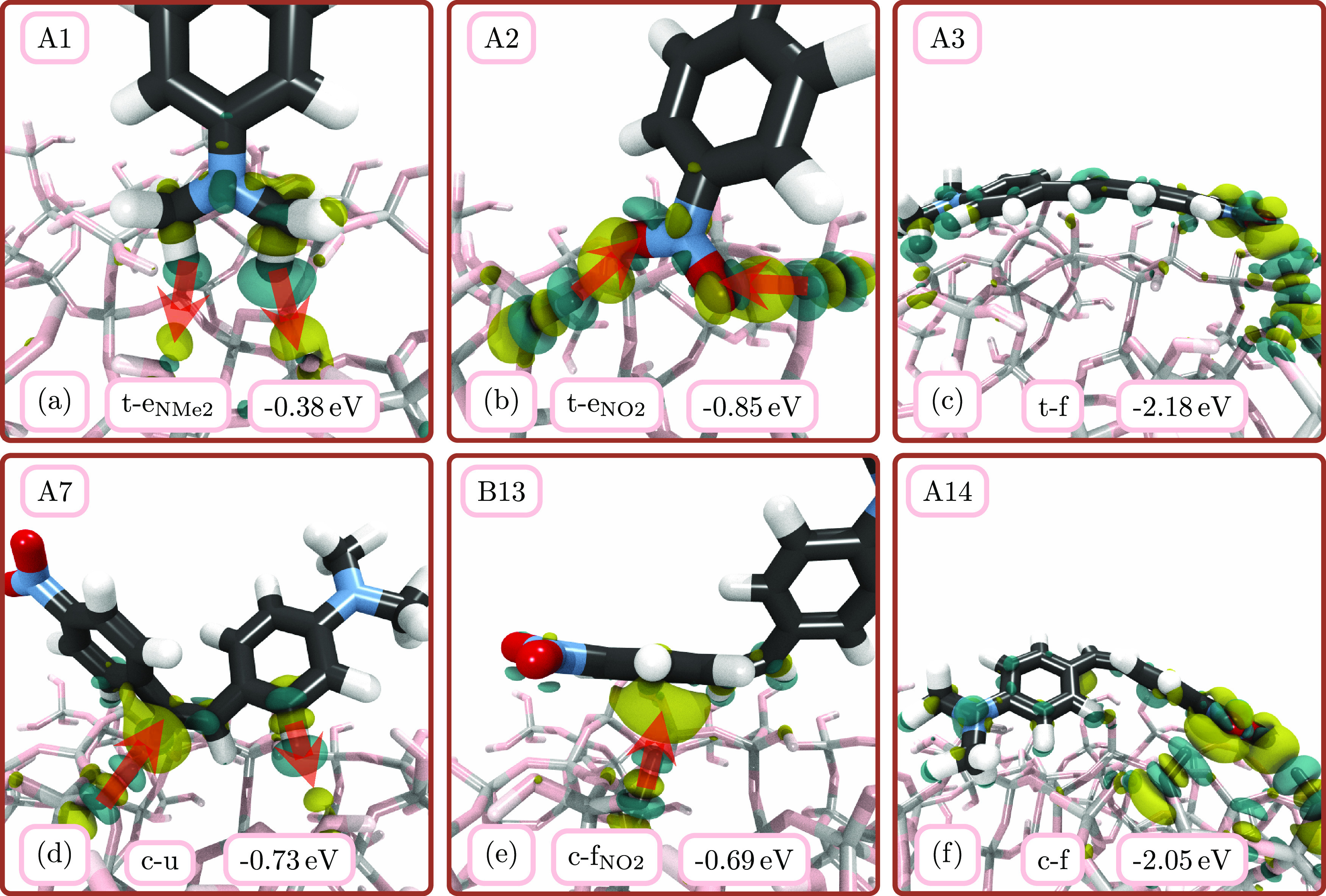
Depiction of representative contacts of DANS with the glass surface (calculations A1, A2, A3, A7, B13, and A14) and corresponding charge density differences for *trans* (a–c) and *cis* (d–f) isomers. Yellow color indicates electron accumulation upon adsorption, whereas blue color indicates electron depletion. Red arrows are used as a guide to the eye to show the direction of electron flow. Next to the orientation labels, we provide the corresponding interaction energies.

Figure 5 illustrates six representative adsorption structures (three *trans* and three *cis*), together with their computed electron density differences according to eq 3. The electron density differences show how the electron cloud of DANS and glass is distorted to form the different noncovalent interactions. The figures also contain the interaction energy (eq 1) of the corresponding structure. All interaction (and adsorption) energies can be found in Tables S8 and S9, and their correlations are plotted in Figure S4.

Figure 5a,b displays two end-on orientations of the *trans* isomer that illustrate the glass–nitro and glass–dimethylamino group interactions without other interactions being present. Their relatively small interaction energies (−0.38 and −0.85 eV) are consistent with the very high relative energies of end-on orientations in Figure 4. In general, interaction energies are correlated well with relative energies (and with adsorption energies), as shown in Figure S4. The depiction in Figure 5a shows two C–H···O bonds (dimethylamino group to glass), which are typically regarded as a weak, special type of HBs.^67^ For each of the two bonds, electronic charge is transferred from the methyl H atoms to the glass O atoms (see the red arrow). Such HBs are typically regarded as very weak HBs,^68^ in line with the small interaction energy. Figure 5b shows two O–H···O bonds (glass to nitro group), where charge is transferred from the OH donor to the O acceptor. These are typical, strong HBs between hetero atoms (O in the present case).^69^ Consequently, the interaction energy is significantly larger than that for the dimethylamino group in Figure 5a. To generalize this comparison between the two functional groups, we computed the average interaction energies for all t-e_NMe2_ and t-e_NO2_ orientations (Table S8), which are −0.32 and −0.85 eV, respectively.

Figure 5c depicts a flat-on *trans* structure (t-f) with one of the largest interaction energies (−2.18 eV). As visible from the geometry and also the charge density difference, here DANS uses the dimethylamino group (left) and the nitro group (right) but also to some extent with the carbon backbone to form multiple noncovalent interactions with the glass. It even appears that DANS bends the conjugated system in order to maximize the number of contacts with the glass, even if the disruption of the conjugated π system also carries an energetic cost. In general, the t-f configuration seems to be highly favored for *trans*, as can be seen from the interaction energies in Table S8. Here, the *trans* structures with interaction energies of more than 2 eV show large deformation energies of DANS, indicating that the molecule has to bend to maximize the interaction energy.

The c-u orientation in Figure 5d is interesting because it does not show any interaction between glass and either of the dimethylamino or nitro groups but nonetheless exhibits a significant interaction energy. On the one hand, this structure presents a C–H···O interaction (from aromatic ring to glass), similar to the dimethylamino group (Figure 5a). On the other hand, there is an unconventional O–H···C interaction^70^ (from glass to a double-bond π system), which is distinct from the cases discussed already. The susceptibility of the double-bond C atoms for such hydrogen bonding might arise because these atoms are partially sp^3^ hybridized due to the disrupted conjugation of the π system in the *cis* isomer. This could be a possible explanation for the relatively high interaction energy of the structure in Figure 5d—much higher than Figure 5a, even though both panels show each two unconventional HBs.

Figure 5e presents a c-f_NO2_ orientation, where the aromatic ring near the nitro group is lying flat on the surface. As indicated by the charge density difference, this structure is mostly stabilized by an O–H···π system (from the glass to the ring) interaction. The structure shows a moderate interaction energy of −0.69 eV, which results not only from the O–H···π system bond but also from weaker nitro–glass and C–H···O interactions. No proper O–H···O HBs toward the nitro group are found, despite the proximity of this group to the surface.

Lastly, Figure 5f shows a *cis* c-fd orientation, which exhibits a large number of contacts, making it the analogue of the *trans* t-f orientation in Figure 5c. The charge density difference reveals multiple interactions—weak C–H···O ones of the dimethylamino group and carbon backbone as well as two very visible O–H···O HBs toward the nitro group. This structure is the *cis* structure with the largest interaction energy, actually the only one above 2 eV for *cis*.

In summary, the analysis in Figure 5 reveals five types of interactions between DANS and the glass surface: (i) C–H···O interactions (from dimethylamino group), (ii) O–H···O interactions (to nitro group), (iii) C–H···O interactions (from aromatic CH), (iv) O–H···C interactions (to C=C group), and (v) O–H···π system interactions (to aromatic rings). By visual inspection of the structures and total interaction energies, it is difficult to extract the relative contributions of each of these individual interactions. Thus, in the next section, we employ multiple linear regression analysis to discriminate among them.

### 3.4 Multiple Linear Regression Analysis

As the previous analyses were qualitative, here we strive to quantify the interaction energies between DANS and the glass surface with the help of multiple linear regression analysis. The dependent (response) variable is the interaction energy. The independent (predictor) variables are the number of contacts separately counted for the five interaction types described above. We count the O–H···π system interactions separately for each of the two aromatic rings, thus arriving at the six predictor variables (i.e., fragments see in Figure S5) mentioned in the computational details: NO_2_, C_6_ (ring near NO_2_), C_2_ (bridge), C_6_ (ring near NMe_2_), NMe_2_, and all nonmethyl H atoms. Further details on these predictor and response variables are given in Tables S10 and S11.

Table 1 presents the final results of the multiple linear regression analysis: the interaction energies per contact for each of the six interaction types, the fitting errors of the interaction energies, and the corresponding significance levels. Note that we discuss the interaction types in a different order as above (see labels i–v). The first four lines in the table (ii, v′, iv, v^″^) refer to interactions where DANS is the HB acceptor (note that DANS does not have hydroxyl groups), while the last two correspond to interactions where DANS is the donor. As can be seen in the first line, the O–H···O HBs between glass and nitro group ii are by far the strongest intramolecular interactions, with more than 0.5 eV per HB. We rationalize the strength of this interaction by the polarity of the nitro group, as evidenced by the Bader charges (see Table S12, atoms 18–20), and by the fact that these are the only “traditional” HBs with noncarbon donor and acceptor.

**Table 1:** Multiple Linear Regression (Intercept of 0) to Estimate the Contribution of Different Parts of the Molecule to the Interaction Energy

label^a^	interaction type	interaction energy per contact/eV	error/eV	significance level^b^
(ii)	O–H···O (NO_2_)	–0.55	0.04	***
(v′)	O–H···π (ring near NO_2_)	–0.18^c^	0.05	**
(iv)	O–H···C (C=C)	–0.23^c^	0.06	***
(v^″^)	O–H···π (ring near NMe_2_)	–0.16^c^	0.04	***
(i)	C–H···O (NMe_2_)	–0.15	0.03	***
(iii)	C–H···O (aromatic CH)	–0.09	0.03	**

a Labels as used in the previous section.

b Significance levels: ’***’ 0.001; ’**’ 0.01; ’*’ 0.05.

c Energy per C atom involved in the contact.

The second to fourth lines of Table 1 (v′, iv, v^″^) refer to the interactions of glass hydroxyl groups with the different parts of the π system of DANS. Here, we note that these contacts can be counted in at least two different ways: counting the hydroxyl groups that are in proximity with the aromatic system (“hydroxyl counting”) or counting the C atoms of the aromatic system that are in proximity to a hydroxyl group (“C atom counting”). For example, Figure 5e shows a single contact according to hydroxyl counting but a triple contact according to C atom counting. In a multiple linear regression based on hydroxyl counting (see Table S13), large errors are obtained for the interaction energy per O–H···π contact due to the large spread in the strength of this interaction. Therefore, we employed C atom counting to obtain the values shown in Table 1. This counting convention leads to smaller errors (and thus a higher significance level), but consequently, the interaction energy is per C atom involved in the contact. Because the O–H···π contacts in our computations involve one to three C atoms, the interaction energies of −0.16 to −0.23 eV per C atom translate into overall O–H···π interaction energies of up to about 0.5 eV. Hence, the unconventional interactions of hydroxyl groups with the π system of DANS can in fact provide a surprisingly large interaction energy contribution—similar in strength to one O–H···O HB.

As shown in the last two lines of Table 1 (i, iii), the weakest intramolecular interactions are provided by the C–H···O contacts (DANS as donor). These include the interactions of the dimethylamino group (−0.15 eV per contact) and of the aromatic CH groups (−0.09 eV per contact). We assume that the former interactions are slightly stronger because the dimethylamino group is more polarized than the aromatic system. This can be seen in the Bader charge analysis (Table S12; NMe_2_: atoms 15–17 and 193–198; aromatic CH: atoms 2–5, 10–13, 183–192).

Furthermore, we performed a separate multiple linear regression analysis for the dispersion contribution to the interaction energy (Δ*E*_int–disp_). These energy contributions are given in Tables S8 and S9, and the multiple linear regression results are given in Table S14. The obtained interaction energies per contact are smaller than in Table 1, with values of −0.06 to −0.17 eV per contact. In particular, Table S14 also lists the percentage contribution of the dispersion interaction energy (relative to the full interaction energy). We find that the conventional HBs of the type O–H···O (NO_2_) have a rather small dispersion contribution of about 30%, showing that the HBs are dominated by electrostatics. On the contrary, all other interactions (v′, iv, v^″^, i, and iii) have a dispersion contribution of 60–80%.

The errors given in Table 1 show that our interaction energies per contact can be regarded as robust. For all six types of interactions, the interaction energies per contact are significantly larger than their respective errors. Hence, it is very unlikely that any of the interaction energies per contact are actually zero (null hypothesis), as indicated by the significance levels. Thus, we find that all of the identified types of contacts play a relevant role in the adsorption of DANS on amorphous silica glass.

## 4 Conclusions

In this study, the adsorption of 4-(*N*,*N*-dimethylamino)-4′-nitrostilbene (DANS) on amorphous silica glass surfaces was investigated. We manually prepared 14 different orientations of *trans*- and *cis*-DANS on four different glass surface models for a total of 56 initial guess geometries. These were optimized by using plane-wave DFT with the D3-PBE functional. The different types of intramolecular interactions and the energetics of the resulting optimized structures were scrutinized to understand the mode of adsorption of DANS onto amorphous silica glass. From the comparison of the obtained optimized structures and the initially prepared geometries, we found that adsorption configurations with only a few molecule–glass contacts are unfavorable because initial structures with few contacts tended to converge to structures with a larger number of contacts. This rules out end-on adsorption for DANS. Instead, adsorption with a large number of contacts was most favorable according to several different flat-on orientations, where both aromatic rings are coming close to the surface. The relative energies of the optimized structures indicated that it is energetically favorable to also bring the nitro group in contact with the surface in addition to the rings. While our optimization strategy does not provide (relative) adsorption free energies, we could still obtain representative structures for the possible modes of adsorption, including limiting cases, i.e., strong and weak interaction cases.

The analysis of the individual optimized structures and their charge density differences identified five different types of intramolecular interactions whose interaction energy contributions we could quantify by means of multiple linear regression analysis. As expected, the nitro group can form O–H···O hydrogen bonds with the hydroxyl groups on the glass surface. These interactions are strong, with an interaction energy gain of about 0.5 eV (12 kcal/mol) per hydrogen bond. More surprisingly, we found that O–H···π and O–H···C interactions provide a significant stabilization of DANS on the glass, with each glass hydroxyl group interacting with a π system providing 0.2–0.5 eV (5–12 kcal/mol). The weakest type of intramolecular interactions is C–H···O interactions, involving either the dimethylamino group or CH bonds of the backbone of DANS. These provide about 0.1 eV (2–3 kcal/mol) per C–H···O bond.

Based on these findings, we expect that DANS would preferably attach the nitro group to the surface while bringing the π system in close contact to the surface. We also found that, in order to maximize contact area, DANS bends notably (especially the *trans* isomer). This rich interaction behavior of DANS could be one of the main reasons for the exceptional sensitivity of its photophysics to the molecular environment. It is known that the nitro group in a push–pull stilbene plays a major role in the excitation process. However, when the electron-withdrawing properties of this group are altered due to hydrogen bonds with the glass surface or when the molecule is bent, the excited states can be significantly shifted in energy. The potential energy surfaces of the excited states can be modified as well, with a likely impact on the photoisomerization and fluorescence behavior. In a future work, we plan to investigate the absorption properties of DANS for the preferred adsorption orientations, as influenced by the different interaction types.

## Supporting Information Available

Depictions of all 56 optimized structures; clustering (definition of parameters, list of parameters, principal components); multiple linear regression (fragment definition, dependent and independent variables, alternative regression analysis, dispersion analysis); and Bader charges (PDF)
All optimized coordinates in VASP format and one INCAR file with input specifications for the calculations (ZIP)
