# Supplementary material for: Adsorption of 4-(N,N-Dimethylamino)-4′-nitrostilbene on an Amorphous Silica Glass Surface
Source: J Phys Chem C Nanomater Interfaces. 2023 Nov 17;127(47):22964–74. doi: 10.1021/acs.jpcc.3c05552 (PMC10694811; doi:10.1021/acs.jpcc.3c05552)
Supplement: Supplementary file 1 — jp3c05552_si_001.pdf [file jp3c05552_si_001.pdf]

Supporting Information: Adsorption of 4-(N,N-Dimethylamino)-4'-Nitrostilbene on an Amorphous Silica Glass Surface

Dóra Vörös,[†,¶] Andrea Angeletti,[‡,¶] Cesare Franchini,[*,‡,§] Sebastian Mai,[*,†] and Leticia González[*,†]

†*Institute of Theoretical Chemistry, Faculty of Chemistry, University of Vienna, Währinger Straße 17, 1090 Vienna, Austria.*

‡*Computational Materials Physics, Faculty of Physics, University of Vienna, Kolingasse 14-16, 1090 Vienna, Austria.*

¶*Vienna Doctoral School in Physics, University of Vienna, Boltzmanngasse 5, 1090 Vienna, Austria.*

§*Department of Physics and Astronomy 'Augusto Righi', Alma Mater Studiorum - Università di Bologna, Bologna, 40127 Italy.*

E-mail: cesare.franchini@univie.ac.at; sebastian.mai@univie.ac.at; leticia.gonzalez@univie.ac.at

Contents

S1 Methods **S-2**

S1.1 Pre-optimization of DANS S-2

S1.2 PBE and RPBE functionals . . . S-2

S1.3 Convergence of k-point and electronic smearing S-3

S2 Results **S-4**

S2.1 Geometric clustering S-6

S2.2 Energetics S-10

S2.3 Multiple linear regression analysis of interaction energies S-13

S1 Methods

S1.1 Pre-optimization of DANS

The cis and trans isomers of DANS were pre-optimized at MP2/cc-pVDZ level of theory before assembling the initial DANS+slab structures. Here, MP2 was chosen because it produced a (slightly) non-planar geometry of trans-DANS, which we used to avoid any possible bias for planar structures during the optimization. For four structures (A6, B3, A13, B9), we investigated the effect of the pre-optimized geometry on the final geometry and interaction energy. To this end, we repeated the optimizations with initial geometries prepared from DANS geometries pre-optimized with PBE-D3, placed at the same positions as in the original calculations. Table S1 demonstrates that the different pre-optimizations do not notably affect the outcomes both in structure and energy. A significantly larger effect can be expected if DANS would be initially positioned differently on the glass. However, as discussed in the main text, sampling the adsorption geometries in such a way goes beyond the scope of our work.

Table S1: Root-mean-square deviations between the optimized structure obtained from pre-optimization with either DFT (PBE/plane wave) or MP2. The interaction energies are given in eV.

Calculation	Starting orientation DFT	End orientation DFT	RMSD (Å)	ΔE_{int} DFT	ΔE_{int} MP2
A6	t-f_{NO2}	t-f	0.074	-2.21	-2.19
B3	t-s_{NMe2}	t-f	0.123	-1.79	-1.77
A13	c-f_{NMe2}	c-fd	0.078	-1.82	-1.82
B9	c-e_{NMe2}	c-d	0.086	-1.22	-1.21

S1.2 PBE and RPBE functionals

To estimate the energy uncertainty due to the choice of functional, we have re-optimized four initial structures, A6, B3, A13, and B9, using the revised Perdew-Burke-Ernzerhof (RPBE)[54] functional, which has been shown to be effective in similar situations, and PBE. The resulting interaction energies are presented in Table S2. It is evident that the two functionals have minor differences, below 0.1 eV. This shows that our results are robust to some extent with respect to the choice of the functional.

Table S2: The table displays the root-mean-square deviation between the structure optimized with RPBE and PBE and the interaction energy respectively. The interaction energies are given in eV.

Calculation	Starting orientation RPBE	End orientation RPBE	RMSD (Å)	ΔE_{int} RPBE	ΔE_{int} PBE
A6	t-f_{NO2}	t-f	0.181	-2.19	-2.19
B3	t-s_{NMe2}	t-f	0.204	-1.85	-1.77
A13	c-f_{NMe2}	c-fd	0.200	-1.83	-1.82
B9	c-e_{NMe2}	c-d	0.087	-1.13	-1.21

S1.3 Convergence of k-point and electronic smearing

We investigated the convergence of the total energies with respect to the k-points grid. We performed single-point calculations on structure A6 using $1 \times 1 \times 1$, $3 \times 3 \times 1$, $5 \times 5 \times 1$, $7 \times 7 \times 1$, and $9 \times 9 \times$, and $11 \times 11 \times 1$ grids. All of these calculations were done with Gaussian smearing. For for a $5 \times 5 \times 1$ grid, we also performed one calculation with the tetrahedron method with Blöchl correction (ISMEAR=-5 in VASP), which is the recommended smearing option for insulators. As shown in Table S3, absolute and interaction energies differ by less than 10 meV and 20 meV, respectively, showing that already a $1 \times 1 \times 1$ and Gaussian smearing produces converged results.

Table S3: Single point energies of the A6 structure using different k-point meshes and smearing options. All calculations use the same geometry and settings described in the main text. ISMEAR 0 stands for Gaussian smearing, while -5 for tetrahedron method with Blöchl corrections.

ISMEAR	k-point	Absolute energy/eV	ΔE_{int}/eV
0	$1 \times 1 \times 1$	-2213.1829	-2.21
0	$3 \times 3 \times 1$	-2213.1740	-2.19
0	$5 \times 5 \times 1$	-2213.1722	-2.20
0	$7 \times 7 \times 1$	-2213.1728	-2.19
0	$9 \times 9 \times 1$	-2213.1830	-2.20
0	$11 \times 11 \times 1$	-2213.1726	-2.19
-5	$5 \times 5 \times 1$	-2213.1825	-2.21

S2 Results

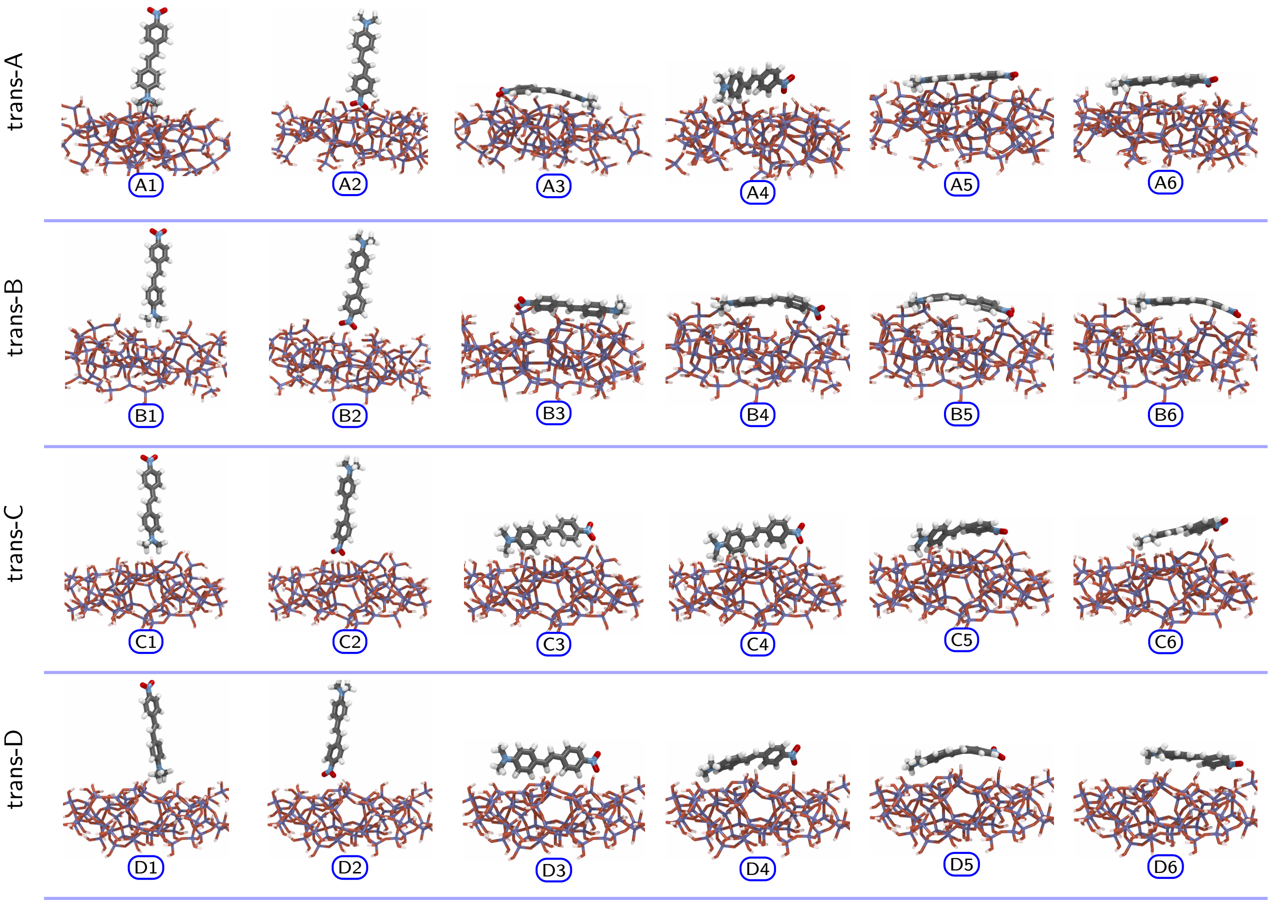

Figure S1: All 24 optimized structures for the trans isomer on glass. Coordinates can be found in the supplementary zip file.

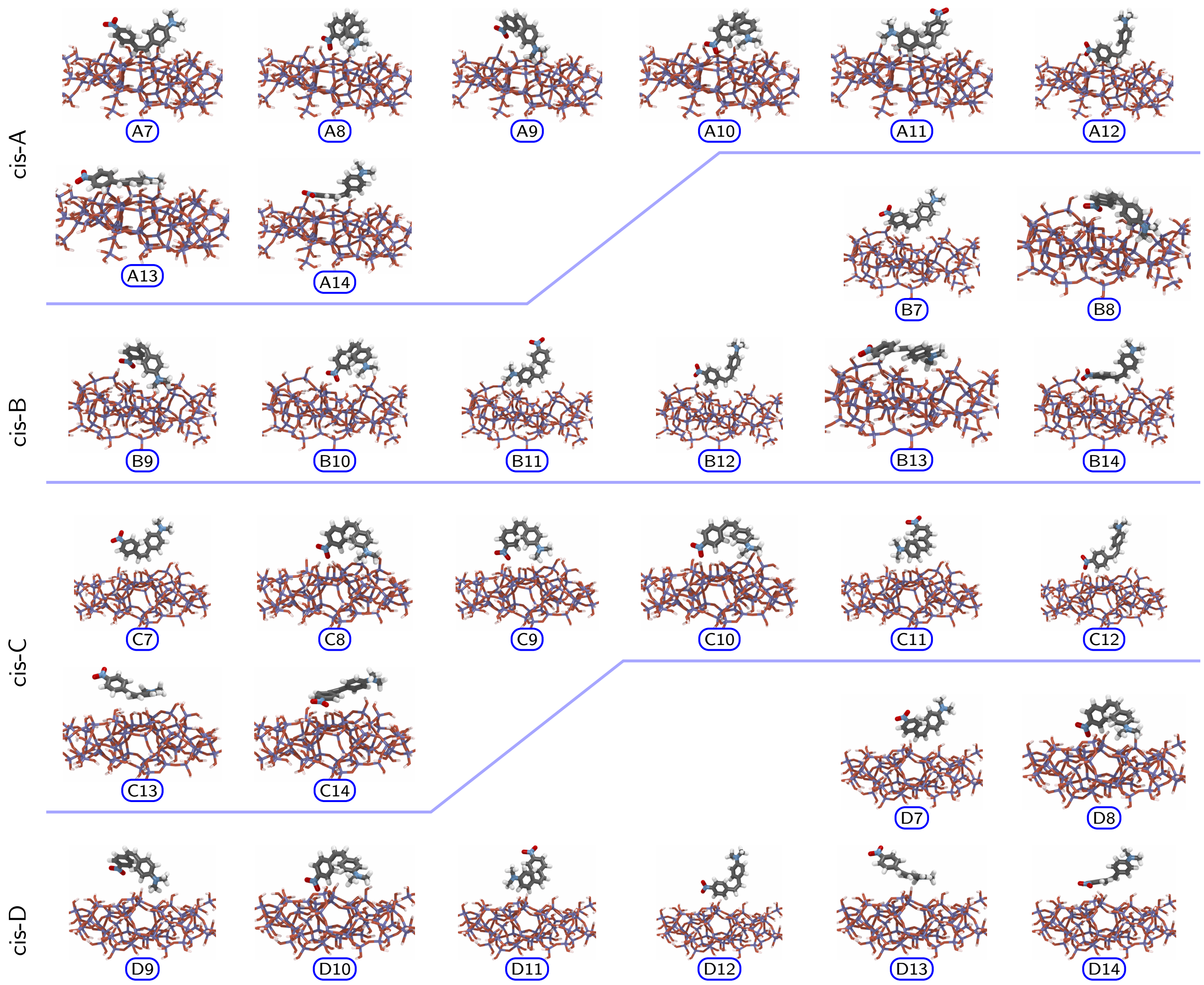

Figure S2: All 32 optimized structures for the cis isomer on glass. Coordinates can be found in the supplementary zip file.

S2.1 Geometric clustering

Figure S3 shows a schematic representation of the data that was collected for the K-Means clustering. The geometric parameters are defined as follows.

- For each of the three NO_2 atoms, we collected the shortest distance to any of the glass O atoms. These three distances were then averaged to obtain the "Distance to NO_2".
- For each of the nine NMe_2 atoms, we collected the shortest distance to any of the glass O atoms. These nine distances were then averaged to obtain the "Distance to NMe_2".
- For the two bridge C atoms, we collected the shortest distance to any of the glass O atoms. These two distances were then averaged to obtain the "Distance to bridge C-C".
- For each of the two aromatic rings, we computed their mean plane and the respective normal vectors. We also computed the mean plane of all glass Si atoms and its normal vector. Then we computed the angles between the two ring normal vectors and the glass normal vector, then averaged these two angles to obtain the "Mean angle of rings".

Table S4 and Table S6 contain these geometric parameters, as they are used for the clustering of the optimized structures. The calculation labels in these tables specify the glass surface (A–D) and the initial orientation (see Figure 2 in the main text).

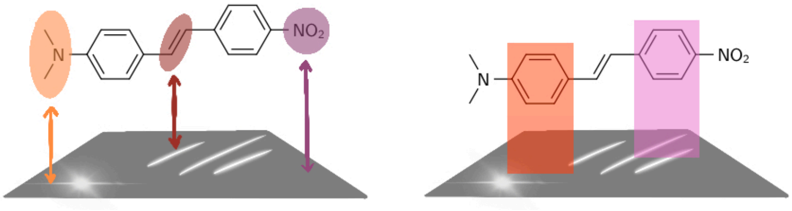

Figure S3: Schematic representation of the geometric parameters that we used for K-Means clustering: "Distance to NO_2" (left, purple), "Distance to NMe_2" (left, orange), "Distance to C-C" (left, red), "Mean angle of rings" (right, mean of the angles indicated in red and purple).

Table S4: Geometrical data used for the K-Mean clustering of the trans isomer on the glass surface. The table includes: the average of the angle closed by the plane of the two aromatic rings with the surface in (Mean angle of rings), the distances of the two functional groups to the surface in Å (Distance to NMe_2 and Distance to NO_2).

Calculation	Starting orientation	End orientation	Mean angle of rings	Distance to NMe_2	Distance to NO_2
A1	t-e_{NMe2}	t-e_{NMe2}	99.03	3.49	15.37
A2	t-e_{NO2}	t-e_{NO2}	81.97	13.90	3.14
A3	t-s_{NMe2}	t-f	15.94	3.45	3.27
A4	t-s_{NO2}	t-s_{NO2}	168.82	4.38	3.90
A5	t-f_{NMe2}	t-f	14.93	3.67	3.33
A6	t-f_{NO2}	t-f	102.97	3.47	3.27
B1	t-e_{NMe2}	t-e_{NMe2}	78.73	3.77	15.54
B2	t-e_{NO2}	t-e_{NO2}	104.40	15.01	3.10
B3	t-s_{NMe2}	t-f	166.12	3.67	3.82
B4	t-s_{NO2}	t-f	157.78	3.52	3.19
B5	t-f_{NMe2}	t-f	41.32	3.59	3.75
B6	t-f_{NO2}	t-f	145.72	3.61	3.26
C1	t-e_{NMe2}	t-e_{NMe2}	78.29	3.46	15.56
C2	t-e_{NO2}	t-e_{NO2}	102.50	14.89	3.04
C3	t-s_{NMe2}	t-s_{NMe2}	138.19	4.85	4.06
C4	t-s_{NO2}	t-s_{NO2}	158.80	4.21	3.76
C5	t-f_{NMe2}	t-f	65.57	3.64	3.33
C6	t-f_{NO2}	t-f	95.61	3.43	4.12
D1	t-e_{NMe2}	t-e_{NMe2}	76.10	3.41	15.47
D2	t-e_{NO2}	t-e_{NO2}	97.48	15.29	3.30
D3	t-s_{NMe2}	t-s_{NMe2}	162.11	5.63	4.24
D4	t-s_{NO2}	t-f	163.44	3.43	4.35
D5	t-f_{NMe2}	t-f	93.47	3.38	4.03
D6	t-f_{NO2}	t-f	142.03	3.81	3.10

Table S5: Loadings[a] of the individual principal components with the geometrical parameters for the trans isomer.

	Mean angle from rings	Distance to NMe_2	Distance to NO_2	Contrib. to total var.
Loading on PC1	-0.56	0.88	-0.55	44%
Loading on PC2	0.75	0.00	-0.76	36%

[a] Loadings: correlations between input geometric parameters and principal component vectors. Values are between +1 (strong correlation) and −1 (strong anticorrelation).

Table S6: Geometrical data used for the K-Mean clustering of the cis isomer on the glass surface. The table includes: the average of the angle closed by the plane of the two aromatic rings with the surface in (Mean angle of rings), the distances of the two functional groups and bridge carbon atoms to the surface in Å (Distance to NMe_2, Distance to NO_2, and Distance to bridge C-C).

Calculation	Starting orientation	End orientation	Mean angle of rings	Distance to NMe_2	Distance to NO_2	Distance to bridge C-C
A7	c-u	c-u	109.69	6.21	6.20	3.02
A8	c-d	c-d	63.43	3.65	3.19	7.99
A9	c-e_{NMe2}	c-d	109.46	3.39	3.89	7.15
A10	c-e_{NO2}	c-d	71.04	3.78	3.14	6.87
A11	c-s_{NMe2}	c-u	66.53	4.87	6.32	2.88
A12	c-s_{NO2}	c-s_{NO2}	73.30	9.26	3.73	3.90
A13	c-f_{NMe2}	c-fd	114.97	3.45	3.41	3.64
A14	c-f_{NO2}	c-f_{NO2}	76.51	7.79	3.80	3.72
B7	c-u	c-u	71.39	8.08	6.10	3.44
B8	c-d	c-d	109.32	3.59	3.06	5.34
B9	c-e_{NMe2}	c-d	67.60	3.57	3.32	6.78
B10	c-e_{NO2}	c-d	71.45	6.10	3.13	7.50
B11	c-s_{NMe2}	c-s_{NMe2}	112.80	4.45	9.32	4.52
B12	c-s_{NO2}	c-s_{NO2}	79.45	10.06	4.20	3.70
B13	c-f_{NMe2}	c-fd	111.66	3.51	3.12	3.70
B14	c-f_{NO2}	c-f_{NO2}	104.56	8.14	3.44	3.40
C7	c-u	c-u	66.08	7.96	7.02	3.62
C8	c-d	c-d	112.38	3.58	3.44	6.02
C9	c-e_{NMe2}	c-d	72.37	3.60	4.12	7.92
C10	c-e_{NO2}	c-d	70.38	3.70	3.86	6.18
C11	c-s_{NMe2}	c-s_{NMe2}	99.65	4.95	9.42	4.48
C12	c-s_{NO2}	c-s_{NO2}	107.11	9.91	4.16	4.92
C13	c-f_{NMe2}	c-f_{NMe2}	102.05	3.56	7.32	3.07
C14	c-f_{NO2}	c-f_{NO2}	102.29	5.35	3.22	3.81
D7	c-u	c-u	57.88	8.44	5.72	3.75
D8	c-d	c-d	112.61	3.45	3.82	5.21
D9	c-e_{NMe2}	c-d	68.11	3.53	6.88	7.12
D10	c-e_{NO2}	c-d	63.84	3.66	3.70	5.96
D11	c-s_{NMe2}	c-s_{NMe2}	109.49	5.18	10.26	4.53
D12	c-s_{NO2}	c-s_{NO2}	109.16	10.73	4.80	4.18
D13	c-f_{NMe2}	c-f_{NMe2}	111.72	4.04	8.66	3.83
D14	c-f_{NO2}	c-f_{NO2}	110.81	8.10	3.63	3.99

Table S7: Loadings[a] of the individual principal components with the geometrical parameters for the cis isomer.

	Mean angle from rings	Distance to NMe_2	Distance to NO_2	Distance to DB	Contrib. to total var.
Loading on PC1	0.56	-0.81	-0.38	-0.81	42%
Loading on PC2	0.52	-0.31	0.82	-0.32	28%

[a] Loadings: correlations between input geometric parameters and principal component vectors. Values are between +1 (strong correlation) and −1 (strong anticorrelation).

S2.2 Energetics

Table S8: Relative energies (ΔE_{rel}), interaction energies (ΔE_{int} see in Eq. (1)), adsorption energies (ΔE_{ads} see in Eq. (2)), deformation energies for molecule (ΔE^{mol}_{def}) and surface (ΔE^{surf}_{def}), and dispersion energies ($\Delta E_{int-disp}$) of the optimized trans structures at four different amorphous surfaces. All energies are given in eV. The relative energies were calculated relative to the most stable structures within A/B (A3) and C/D (D4) separately.

Calculation	Starting orientation	End orientation	ΔE_{rel}	ΔE_{int}	ΔE_{ads}	ΔE^{mol}_{def}	ΔE^{surf}_{def}	$\Delta E_{int-disp}$
A1	t-e_{NMe2}	t-e_{NMe2}	1.60	-0.38	-0.74	-0.03	0.20	-0.24
A2	t-e_{NO2}	t-e_{NO2}	1.17	-0.85	-1.17	-0.06	0.18	-0.31
A3	t-s_{NMe2}	t-f	0.00	-2.18	-2.34	-0.13	0.10	-1.14
A4	t-s_{NO2}	t-s_{NO2}	0.59	-1.48	-1.75	-0.11	0.18	-0.64
A5	t-f_{NMe2}	t-f	0.54	-1.63	-1.80	-0.06	0.04	-0.81
A6	t-f_{NO2}	t-f	0.15	-2.19	-2.18	-0.13	-0.07	-1.00
B1	t-e_{NMe2}	t-e_{NMe2}	1.73	-0.28	-0.61	-0.04	0.17	-0.16
B2	t-e_{NO2}	t-e_{NO2}	1.45	-1.02	-0.88	-0.09	-0.24	-0.22
B3	t-s_{NMe2}	t-f	0.58	-1.77	-1.76	-0.10	-0.10	-0.85
B4	t-s_{NO2}	t-f	0.57	-2.23	-1.77	-0.17	-0.48	-1.09
B5	t-f_{NMe2}	t-f	1.08	-1.42	-1.26	-0.10	-0.25	-0.69
B6	t-f_{NO2}	t-f	0.65	-1.94	-1.69	-0.10	-0.34	-1.01
C1	t-e_{NMe2}	t-e_{NMe2}	1.15	-0.31	-0.48	-0.04	0.01	-0.25
C2	t-e_{NO2}	t-e_{NO2}	0.81	-0.91	-0.81	-0.05	-0.24	-0.21
C3	t-s_{NMe2}	t-s_{NMe2}	0.39	-1.22	-1.24	-0.06	-0.11	-0.63
C4	t-s_{NO2}	t-s_{NO2}	0.18	-1.65	-1.44	-0.17	-0.23	-0.81
C5	t-f_{NMe2}	t-f	0.02	-1.82	-1.61	-0.13	-0.27	-0.91
C6	t-f_{NO2}	t-f	0.20	-1.42	-1.43	-0.05	-0.13	-0.80
D1	t-e_{NMe2}	t-e_{NMe2}	1.04	-0.30	-0.59	-0.03	0.13	-0.29
D2	t-e_{NO2}	t-e_{NO2}	0.89	-0.61	-0.73	-0.06	-0.02	-0.19
D3	t-s_{NMe2}	t-s_{NMe2}	0.46	-0.95	-1.17	-0.04	0.07	-0.52
D4	t-s_{NO2}	t-f	0.00	-1.50	-1.62	-0.09	0.02	-0.87
D5	t-f_{NMe2}	t-f	0.07	-1.50	-1.55	-0.12	-0.02	-0.84
D6	t-f_{NO2}	t-f	0.01	-1.44	-1.61	-0.04	0.02	-0.81

Table S9: Relative energies (ΔE_{rel}), interaction energies (ΔE_{int} see in Eq. (1)), adsorption energies (ΔE_{ads} see in Eq. (2)), deformation energies for molecule (ΔE^{mol}_{def}) and surface (ΔE^{surf}_{def}), and dispersion energies ($\Delta E_{int-disp}$) of the optimized trans structures at four different amorphous surfaces. All energies are given in eV. The relative energies were calculated relative to the most stable structures within A/B (B13) and C/D (C8) separately.

Calculation	Starting orientation	End orientation	ΔE_{rel}	ΔE_{int}	ΔE_{ads}	ΔE^{mol}_{def}	ΔE^{surf}_{def}	$\Delta E_{int-disp}$
A7	c-u	c-u	1.09	-0.73	-1.04	-0.03	0.15	-0.61
A8	c-d	c-d	0.74	-1.07	-1.39	-0.05	0.18	-0.40
A9	c-e_{NMe2}	c-d	0.78	-1.15	-1.35	-0.05	0.05	-0.51
A10	c-e_{NO2}	c-d	0.84	-1.11	-1.28	-0.09	0.07	-0.48
A11	c-s_{NMe2}	c-u	0.82	-1.01	-1.31	-0.04	0.15	-0.72
A12	c-s_{NO2}	c-s_{NO2}	0.88	-0.82	-1.25	-0.06	0.30	-0.32
A13	c-f_{NMe2}	c-fd	0.22	-1.82	-1.91	-0.11	0.01	-0.61
A14	c-f_{NO2}	c-f_{NO2}	1.13	-0.69	-0.99	-0.02	0.13	-0.56
B7	c-u	c-u	1.19	-0.60	-0.94	-0.02	0.16	-0.42
B8	c-d	c-d	0.57	-1.40	-1.56	-0.07	0.04	-0.79
B9	c-e_{NMe2}	c-d	0.77	-1.21	-1.36	-0.09	0.06	-0.48
B10	c-e_{NO2}	c-d	0.74	-1.19	-1.38	-0.11	0.12	-0.29
B11	c-s_{NMe2}	c-s_{NMe2}	1.19	-0.66	-0.94	-0.04	0.13	-0.45
B12	c-s_{NO2}	c-s_{NO2}	1.03	-0.89	-1.09	-0.04	0.06	-0.37
B13	c-f_{NMe2}	c-fd	0.00	-2.05	-2.12	-0.09	-0.02	-0.90
B14	c-f_{NO2}	c-f_{NO2}	0.78	-1.18	-1.34	-0.08	0.05	-0.67
C7	c-u	c-u	1.09	-0.32	-0.51	-0.01	0.01	-0.27
C8	c-d	c-d	0.00	-1.86	-1.60	-0.17	-0.28	-0.58
C9	c-e_{NMe2}	c-d	0.77	-0.90	-0.83	-0.11	-0.15	-0.39
C10	c-e_{NO2}	c-d	0.53	-1.09	-1.07	-0.05	-0.16	-0.48
C11	c-s_{NMe2}	c-s_{NMe2}	0.94	-0.46	-0.67	-0.01	0.02	-0.37
C12	c-s_{NO2}	c-s_{NO2}	0.90	-0.67	-0.70	-0.04	-0.12	-0.35
C13	c-f_{NMe2}	c-f_{NMe2}	0.65	-0.82	-0.95	-0.03	-0.02	-0.55
C14	c-f_{NO2}	c-f_{NO2}	0.11	-1.59	-1.50	-0.08	-0.20	-0.66
D7	c-u	c-u	0.89	-0.45	-0.71	-0.01	0.08	-0.35
D8	c-d	c-d	0.28	-1.48	-1.32	-0.16	-0.19	-0.71
D9	c-e_{NMe2}	c-d	0.94	-0.41	-0.67	-0.02	0.09	-0.32
D10	c-e_{NO2}	c-d	0.36	-1.35	-1.25	-0.13	-0.15	-0.55
D11	c-s_{NMe2}	c-s_{NMe2}	0.95	-0.42	-0.66	-0.03	0.09	-0.33
D12	c-s_{NO2}	c-s_{NO2}	0.98	-0.35	-0.62	-0.03	0.12	-0.30
D13	c-f_{NMe2}	c-f_{NMe2}	0.66	-0.73	-0.94	-0.03	0.05	-0.44
D14	c-f_{NO2}	c-f_{NO2}	0.59	-0.92	-1.01	-0.07	-0.03	-0.42

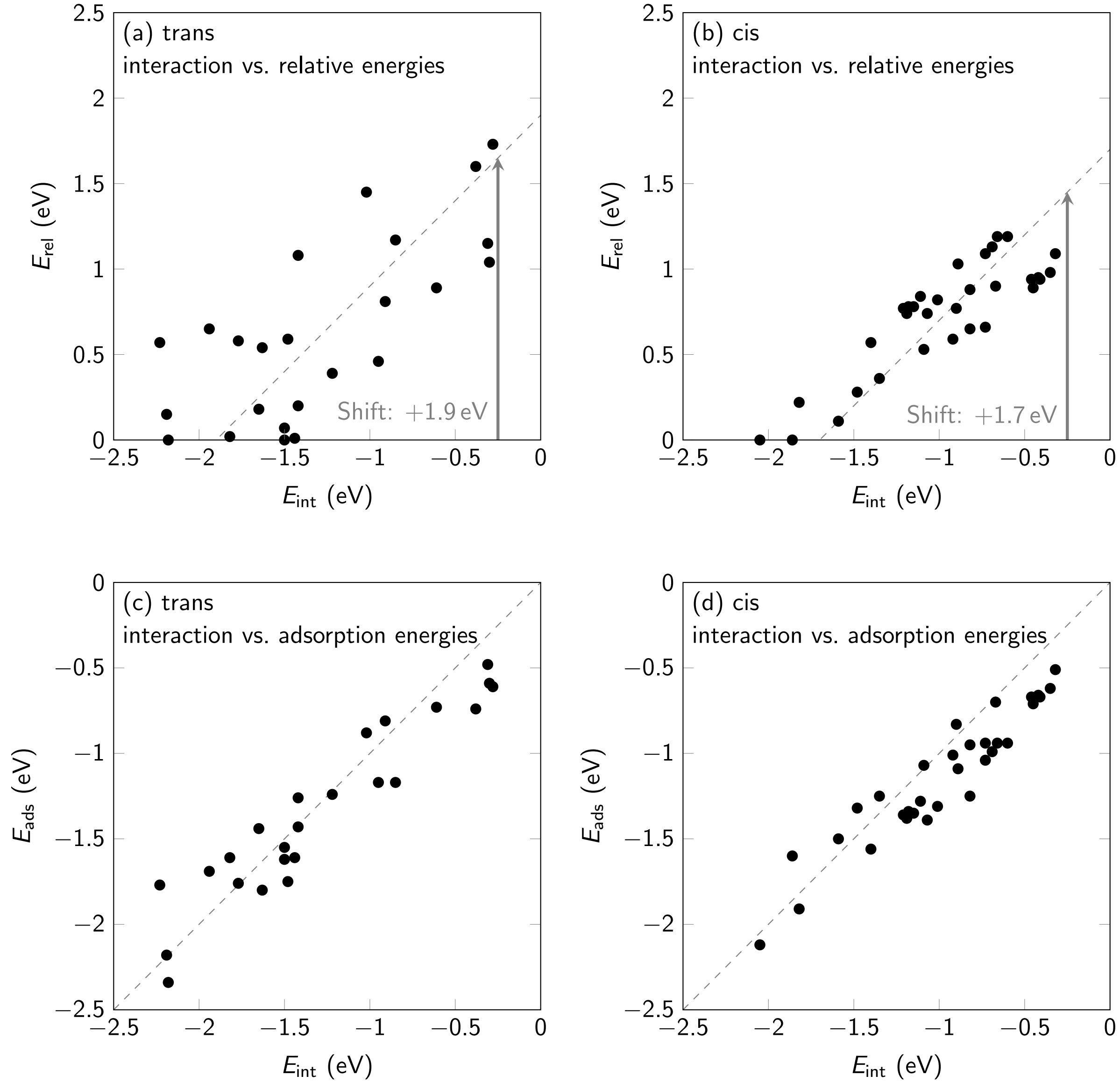

Figure S4: The correlation between interaction energy and relative energy (shown in panels a and b) and between interaction energy and adsorption energy (depicted in panels c and d) is demonstrated by plotting them against each other. Note that the relative energies are shifted due to the way we define them.

S2.3 Multiple linear regression analysis of interaction energies

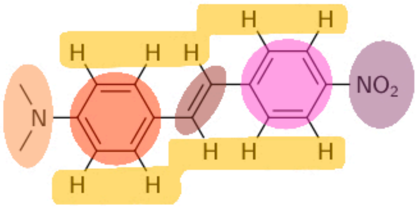

Figure S5: The fragmentation scheme that was used to count the contacts between fragments of DANS and the glass surface.

Table S10: Counting of the different type of contacts/interactions of the trans-DANS with glass. The table includes: name of individual calculation; orientation after optimization; contacts with NO_2, contacts with the aromatic rings and the bridge carbon atoms, contacts with NMe_2; contacts with the hydrogen atoms of the π system.

Calculation	End orientation	Interaction energies	NO_2	NO_2 ring[a]	bridge C-C[a]	NMe_2 ring[a]	NMe_2	π C-H
A1	t-e_{NMe2}	-0.38	0	0	0	0	2	0
A2	t-e_{NO2}	-0.85	2	0	0	0	0	0
A3	t-f	-2.18	2	0	0	3	3	2
A4	t-s_{NMe2}	-1.48	1	0	0	3	0	1
A5	t-f	-1.63	1	0	0	0	3	0
A6	t-f	-2.19	2	1	0	1	1	1
B1	t-e_{NMe2}	-0.28	0	0	0	0	1	0
B2	t-e_{NO2}	-1.02	2	0	0	0	0	0
B3	t-f_{close}	-1.77	1	0	0	0	1	1
B4	t-f_{close}	-2.23	2	3	0	0	3	0
B5	t-f	-1.42	1	0	2	1	2	0
B6	t-f	-1.94	1	2	1	0	3	0
C1	t-e_{NMe2}	-0.31	0	0	0	0	3	0
C2	t-e_{NO2}	-0.91	2	0	0	0	0	0
C3	t-s_{NMe2}	-1.22	1	0	0	1	1	2
C4	t-s_{NMe2}	-1.65	2	0	0	2	1	1
C5	t-e_{close}	-1.82	2	0	0	3	3	0
C6	t-f	-1.42	1	2	0	1	3	0
D1	t-e_{NMe2}	-0.30	0	0	0	0	3	0
D2	t-e_{NO2}	-0.61	1	0	0	0	0	0
D3	t-s_{NMe2}	-0.95	1	0	0	0	0	4
D4	t-e_{close}	-1.50	1	1	2	1	2	0
D5	t-f	-1.50	1	1	2	1	2	0
D6	t-f	-1.44	1	1	1	1	2	0

[a] Using "C atom counting", see main text.

Table S11: Counting of the different type of contacts/interactions of the cis-DANS with glass. The table includes: name of individual calculation; orientation after optimization; contacts with NO_2, contacts with the aromatic rings and the bridge carbon atoms, contacts with NMe_2; contacts with the hydrogen atoms of the π system.

Calculation	End orientation	Interaction energies	NO_2	NO_2 ring[a]	bridge C-C[a]	NMe_2 ring[a]	NMe_2	π C-H
A7	c-u	-1.07	1	0	0	0	3	0
A8	c-d	-0.73	0	1	1	0	0	2
A9	c-d	-1.15	1	0	0	0	3	1
A10	c-d	-1.11	2	0	0	0	2	0
A11	c-u	-1.01	0	0	1	2	0	0
A12	c-s_{NO2}	-0.82	1	1	0	0	0	3
A13	c-f	-1.82	1	0	0	3	3	2
A14	c-f_{NO2}	-0.69	0	2	0	0	0	2
B7	c-u	-0.60	0	2	1	0	0	4
B8	c-d	-1.40	1	0	0	0	2	1
B9	c-d	-1.21	1	0	0	0	3	1
B10	c-e_{NO2}	-1.19	2	0	0	0	0	1
B11	c-s_{NMe2}	-0.66	0	0	0	1	1	2
B12	c-s_{NO2}	-0.89	1	1	0	0	0	1
B13	c-f	-2.05	2	1	1	0	3	0
B14	c-f_{NO2}	-1.18	1	0	2	0	0	0
C7	c-u	-0.32	0	0	0	0	0	2
C8	c-d	-1.86	2	0	0	0	3	2
C9	c-d	-0.90	1	0	0	0	3	0
C10	c-d	-1.09	1	0	0	0	2	1
C11	c-s_{NMe2}	-0.46	0	0	0	0	1	3
C12	c-s_{NO2}	-0.67	1	1	0	0	0	0
C13	c-f_{NMe2}	-0.82	0	0	1	2	3	1
C14	c-f	-1.59	2	0	0	3	0	0
D7	c-u	-0.45	0	2	0	0	0	4
D8	c-d	-1.48	2	0	0	2	3	1
D9	c-e_{NMe2}	-0.41	0	0	0	0	3	0
D10	c-d	-1.35	2	0	0	1	3	2
D11	c-s_{NMe2}	-0.42	0	0	0	1	0	1
D12	c-s_{NO2}	-0.35	0	0	0	0	0	2
D13	c-f_{NMe2}	-0.73	0	0	0	3	2	0
D14	c-f_{NO2}	-0.92	1	1	1	0	0	0

[a] Using “C atom counting”, see main text.

Table S12 displays the Bader charges of DANS during various types of adsorption in distinct geometries from Fig. 5, which were calculated using the Bader Charge Analysis code developed by the Henkelman group.

Table S12: Bader charges on the atoms of DANS in different adsorption structures from Fig. 5 (A1, A2, A3, A7, B13, and A14).

		A1 t-e_{NMe2}	A2 t-e_{NO2}	A3 t-f	A7 c-u	B13 c-f	A14 c-f_{NO2}
C	1	0.20	0.20	0.30	0.12	0.29	0.22
C	2	-0.03	-0.08	-0.09	-0.03	-0.13	-0.12
C	3	-0.10	0.02	0.00	0.00	0.03	-0.02
C	4	-0.05	-0.02	-0.09	-0.07	-0.11	-0.05
C	5	-0.04	0.08	0.04	0.04	0.01	-0.06
C	6	0.06	-0.08	-0.01	-0.05	-0.06	-0.01
C	7	-0.06	0.03	-0.04	-0.18	0.01	-0.03
C	8	-0.08	-0.13	-0.05	-0.04	-0.13	-0.15
C	9	-0.02	-0.03	0.09	0.01	-0.09	0.02
C	10	-0.01	-0.05	-0.11	-0.02	0.09	-0.09
C	11	-0.01	-0.06	-0.06	-0.11	-0.20	-0.11
C	12	-0.13	-0.04	0.00	-0.12	-0.15	-0.01
C	13	-0.10	-0.10	-0.06	-0.05	0.10	-0.01
C	14	0.41	0.40	0.43	0.37	0.37	0.30
C	15	0.16	0.25	0.20	0.20	0.20	0.23
C	16	0.22	0.23	0.17	0.26	0.22	0.25
N	17	-1.15	-1.13	-1.11	-1.14	-1.15	-1.20
N	18	0.20	0.17	0.11	0.21	0.11	0.21
O	19	-0.44	-0.46	-0.51	-0.43	-0.48	-0.41
O	20	-0.44	-0.44	-0.49	-0.41	-0.46	-0.41
H	183	0.14	0.15	0.14	0.15	0.15	0.15
H	184	0.14	0.12	0.10	0.15	0.16	0.13
H	185	0.08	0.08	0.09	0.12	0.13	0.11
H	186	0.05	0.09	0.08	0.08	0.12	0.15
H	187	0.09	0.06	0.06	0.10	0.06	0.09
H	188	0.04	0.10	0.08	0.07	0.07	0.12
H	189	0.08	0.09	0.09	0.12	0.04	0.07
H	190	0.08	0.08	0.03	0.10	0.13	0.09
H	191	0.08	0.07	0.07	0.10	0.09	0.08
H	192	0.07	0.10	0.08	0.07	0.08	0.08
H	193	0.08	0.06	0.06	0.02	0.10	0.07
H	194	0.11	0.04	0.10	0.10	0.08	0.04
H	195	0.08	0.04	0.05	0.08	0.09	0.04
H	196	0.08	0.03	0.13	0.03	0.07	0.08
H	197	0.07	0.05	0.07	0.03	0.10	0.02
H	198	0.06	0.09	0.06	0.09	0.05	0.06

Table S13 shows an alternative multiple linear regression analysis of the interaction energies using "hydroxyl counting" for the O–H$\cdots\pi$ and O–H$\cdots C$ interaction types (see discussion in the main text). This method, however, results in larger errors and thus lower significance levels. These larger errors can be attributed to the spread in interaction energy when a larger or smaller part of the π-system is involved in the contact.

Table S13: Multiple linear regression (intercept of 0) to estimate the contribution of different parts of the molecule to the interaction energy using "hydroxyl counting".

Label[a]	Interaction type	Interaction energy per contact/eV	Error /eV	Significance level[b]
(ii)	O–H$\cdots$O (NO_2)	-0.57	0.05	***
(v′)	O–H$\cdots\pi$ (ring near NO_2)	-0.20	0.10	*
(iv)	O–H$\cdots$C (C=C)	-0.28	0.11	*
(v″)	O–H$\cdots\pi$ (ring near NMe_2)	-0.27	0.09	**
(i)	C–H$\cdots$O (NMe_2)	-0.16	0.03	***
(iii)	C–H$\cdots$O (aromatic CH)	-0.10	0.03	**

[a] Labels as used in the previous section.
[b] Significance levels: '***' 0.001; '**' 0.01; '*' 0.05.

We carried out a multiple linear regression analysis of the dispersion contribution to the interaction energies ($\Delta E_{\text{int-disp}}$, see Tables S8 and S9). To calculate $\Delta E_{\text{int-disp}}$, we applied equation (1) to the D3 dispersion energies. The results are shown in Table S14, demonstrating that the dispersion energy contribution associated with conventional hydrogen bonding (O–H$\cdots$O (NO_2)) is smaller in comparison to the other interactions.

Table S14: Multiple linear regression (intercept of 0) to estimate the contribution of different parts of the molecule to the dispersion interaction energy. The proportion of the dispersion interaction energy relative to the total interaction energy is indicated by the percentage.

Label[a]	Interaction type	Interaction energy per contact/eV	Error /eV	Significance level[b]
(ii)	O–H$\cdots$O (NO_2)	-0.17 (31%)	0.03	***
(v′)	O–H$\cdots\pi$ (ring near NO_2)	-0.14[c] (78%)	0.03	***
(iv)	O–H$\cdots$C (C=C)	-0.17[c] (74%)	0.04	***
(v″)	O–H$\cdots\pi$ (ring near NMe_2)	-0.10[c] (63%)	0.02	***
(i)	C–H$\cdots$O (NMe_2)	-0.09 (60%)	0.02	***
(iii)	C–H$\cdots$O (aromatic CH)	-0.06 (67%)	0.02	**

[a] Labels as used in the previous section.
[b] Significance levels: '***' 0.001; '**' 0.01; '*' 0.05.
[c] Energy per C atom involved in the contact.
